# The effect of combining PD-1 agonist and low-dose Interleukin-2 on treating systemic lupus erythematosus

**DOI:** 10.3389/fimmu.2023.1111005

**Published:** 2023-03-08

**Authors:** Bing Wang, Can Chen, Xia Liu, Shuang Zhou, Ting Xu, Min Wu

**Affiliations:** ^1^ Department of Rheumatology and Immunology, The Third Affiliated Hospital of Soochow University, Changzhou, Jiangsu, China; ^2^ Department of Oncology, The Third Affiliated Hospital of Soochow University, Changzhou, Jiangsu, China

**Keywords:** PD-1 agonist, systemic lupus erythematosus, targeted therapy, pathogenesis, biomarker

## Abstract

Systemic lupus erythematosus (SLE) is a chronic autoimmune disease involving multiple organs. It is often called “immortal cancer” due to the difficulties in disease treatment. As the cornerstone of immune regulation, the programmed cell death protein 1 (PD-1) has been extensively studied in the context of chronic inflammation due to its ability of regulating immune response and immunosuppression. Recently, more and more studies on rheumatic immune related complications have also focused on PD-1 and proposed that the use of PD-1 agonist could inhibit the activation of lymphocytes and alleviate SLE disease activity. In this review, we summarized the role of PD-1 in SLE, implicating its potential application as a biomarker to predict SLE disease activity; we also proposed that the combination of PD-1 agonist and low-dose IL-2 may have better therapeutic efficacy, shining light on a new direction for developing specific treatment approaches.

## 1 Introduction

Systemic lupus erythematosus (SLE) is a chronic inflammatory autoimmune disease, which can affect multiple systems of the body and has high heterogeneity (1, 2). The pathogenesis of SLE involves abnormal activation of immune system and, lymphocytes, which then proliferate and attack various host tissues (3–5). Programmed death factor 1 and its ligand (PD-1/PD-L1) negative costimulatory molecules belong to the CD28/B7 family (6–8). PD-1 was initially found in the 2B4.11 clone of apoptotic mouse T cell hybridoma, and its structure was similar to that of CD28 molecule (1). Subsequent clinical trials on SLE showed that PD-1 is present on various immune cells and plays a role in inhibiting the activation of patients’ own lymphocytes (9, 10). The regulatory T (Treg) cells and effector T (Teff) cells regulate each other to maintain immune homeostasis (11). The loss of homeostatic balance or Treg function is associated with overactivated Teff, which responses against self-antigens and, causes autoimmune disease. As a famous co-signaling receptor, PD-1 plays an indispensable role in regulating the proliferation and activation of Teff and Treg cells, and thus it is involved in the development of autoimmune diseases (12). Recently, some studies showed that sPD-1(the soluble form of PD-1)played a role in autoimmune inflammation (13). Compared with other negative regulators with non-antigen specific characteristics, the absence of PD-1 can affect antigen specific autoimmune response (14). The expression levels of PD-1 and sPD-1 are significantly associated with the pathogenesis of SLE, and they can serve as independent biomarkers and prognostic factors of the disease progression (15–17). So far, the mechanisms of SLE pathogenesis are still unclear. This article reviewed the relationship between PD-1 and SLE, and proposed to use PD-1 as a predictive biomarker for SLE disease activity. We also discussed the potential of combining PD-1 agonist and low-dose interleukin-2 for the treatment of SLE and how this regimen might improve clinical outcomes. Finally, we explored the problem of off-target effect and the biosafety of targeted immune therapy.

## 2 Structure and biological function of PD-1

As a negative co-inhibitory receptor, PD-1(CD279) was originally identified in mice with different autoimmune phenotypes. It regulates the antigen reaction threshold of T cells and B cells in peripheral blood to balance immunity. PD-1 is an important member of the CD28/B7 family and the immunoglobulin superfamily, with 288 amino acid residues. It mainly exists in activated T lymphocytes, B lymphocytes, NK cells, monocytes, and dendritic cells. PD-1 is composed of an N-terminal IgV domain, a transmembrane domain, a cytoplasmic tail with two tyrosine-based signal motifs, and an 20-amino acids side chain. The two tyrosine-based signal sequences form a tyrosine-based immune receptor switch motif (ITSM) that ensures PD-1 performs its inhibitory function (6). PD-1 can suppress harmful immune reactions and maintain immune tolerance by blocking immune checkpoints.

PD-1 plays an inhibitory role in cellular immunity, humoral immunity and innate immunity. In cellular immunity, the activation of PD-1 pathway leads to the phosphorylation of its cytoplasmic domain tyrosine and the recruitment of homology 2-domain-containing protein tyrosine phosphatase (SHP)-2 at the tyrosine site. This cascade of SHP-2 recruitment then leads to the dephosphorylation of ZAP70 and inhibits the production of downstream interleukin (IL)-2, ultimately inducing T cell anergy. In humoral immunity, PD-1 pathway activation and B cell receptor signaling produce co-stimulation, which results in SHP-2 recruitment to these phosphorylated regions of PD-1 protein, leading to the inactivation of downstream molecules and inducing B cell anergy. In innate immunity, the activation of PD-1 pathway can inhibit the secretion of inflammatory factors and inhibit the function of NK cells (18).

There are two kinds of PD-1 ligands, PD-L1 and PD-L2. It is generally believed that the distribution of PD-L1 is much wider than that of PD-L2 (19). PD-L1 is also a type I transmembrane protein, which can interact with PD-1 through the extracellular domain, leading to the conformational change of PD-1 (20). When PD-1 binds to PD-L1, it can regulate the peripheral tolerance of immune cells and arrest T cells at G0/G1 phase.

**Figure 1:**
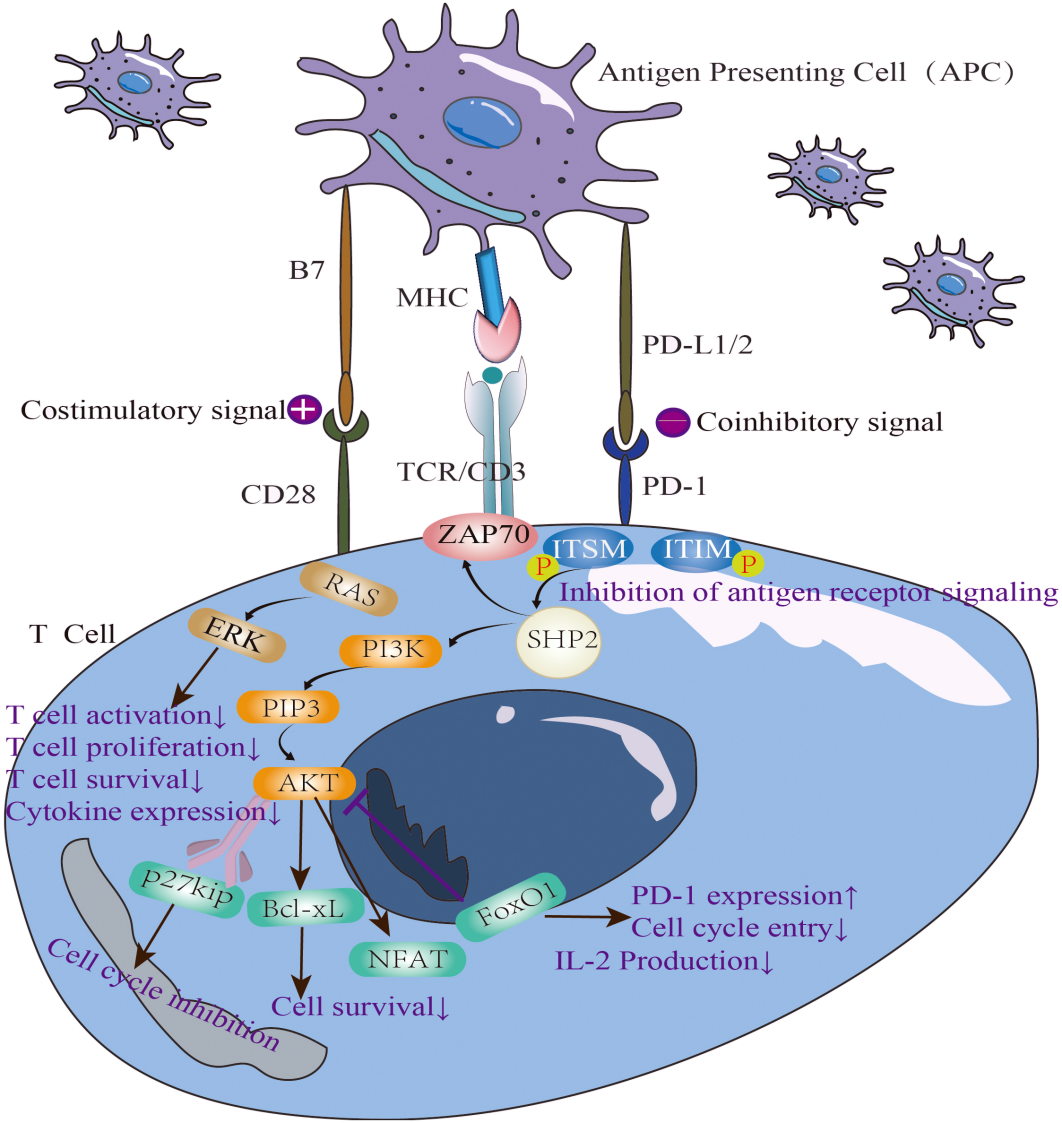
The immunosuppressive mechanism of PD-1/PD-L1 pathway and the negative correlation between PD-1 and IL-2.

In recent years, the research on PD-1 immune checkpoint has focused on its transcriptional and, post-transcriptional regulation. The high expression of PD-1 is a marker of the gradual loss of T cell function and T cell depletion (20). PD-1 releases sPD-L1 under the catalysis of matrix metalloproteinase. sPD-L1 can not only reflect the expression of PD-1, but also block the response of PD-1 to PD-L1 (21, 22). There is a significant overlap in genetic susceptibility between SLE and type 1 diabetes (T1D) (23), by using a nonobese diabetic (NOD)mice model, Mohammed Javeed I. Ansari et al. demonstrated that after PD-1–PD-L1 blockade, GAD-reactive T cells were in a highly active state and targeted host tissues, leading to increased destruction of self-organization (**Figure 1**). The sensitivity of Teff cells to PD-1-PD-L1 inhibitory pathway, provides a promising strategy of immunotherapy. Moreover, it is discovered that the parenchymal cells might down-regulate lymphocyte function at the inflammatory site (24). The membrane bound PD-1 protein can be hydrolyzed and released as the soluble PD-1 (sPD-1) (17), and recent studies have reported that the expression of sPD-1 is well correlated with the activity of autoimmune diseases (25).

## 3 Mechanism and implications of PD-1/PD-L1 axis

PD-1/PD-L1 both have soluble and membrane bound types. The soluble molecules are similar to cytokines and function through the circulatory system, while the membrane bound molecules participate in immune regulation by interacting with other membrane type molecules. The main molecular forms of PD-1/PD-L1 axis are mPD-1 and mPD-L1. The immunogenicity of antigen receptors often results in incomplete thymic negative selection, which can prevent autoimmune reactions that attack host tissues, and PD-1/PD-L1 axis plays a key role in this process.

Taku Okazaki et al. reported that when the co-inhibitory molecule PD-L1 is deficient, the threshold of antigen recognition reaction in T cells decreased, and the CD4^+^lymphocyte activity increased, which led to increased sensitivity of Teff to antigens (26). Shu Zhang et al. also found that the genetic polymorphism of PD-1/PD-L1 pathway was related to the genetic susceptibility to rheumatic diseases. Contrary to PD-1, the polymorphism of PD-L1 gene seems to have no clear relationship with SLE (27), but the lack of PD-L1 is related to increased disease activity (27). Moreover, it has been shown that the manipulation of PD-1/PD-L1 pathway can significantly alter disease severity in multiple animal models (18, 28).

**Figure 2:**
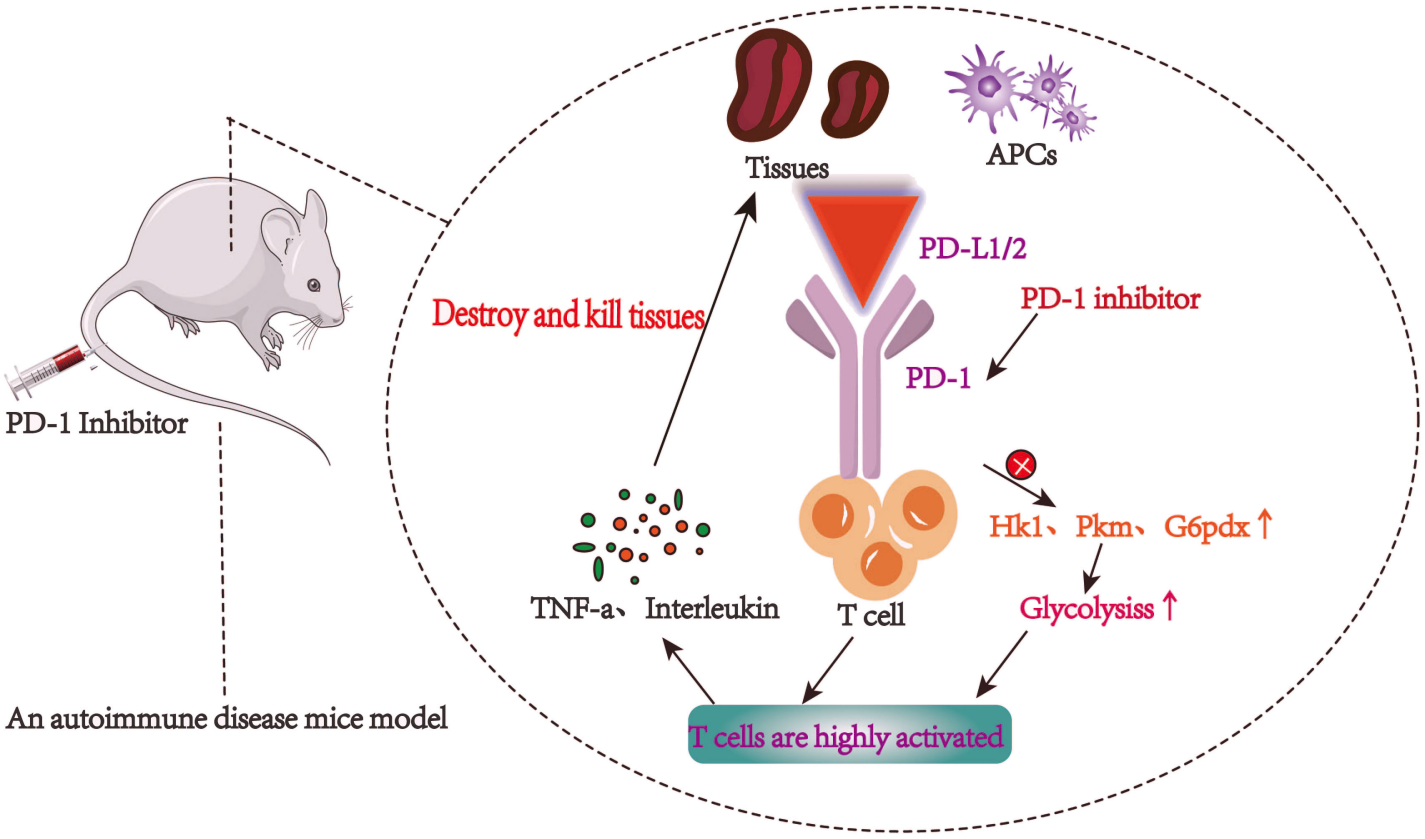
T cells are highly active and autoimmune inflammatory reaction is intensified after PD-1/PD-L1 pathway is blocked.

PD-1/PD-L1 axis has been an important target of immunotherapy for many years, especially in the treatment of autoimmune diseases (29). PD-1 is overexpressed on the surface of activated T cells, which leads to the release of inflammatory factors, such as tumor necrosis factor-a (TNF-a) and interleukin. These inflammatory factors then result in PD-L1 overexpression in tissues, which in turn inhibits the activation of T cells (30, 31). An important sign of T cell activation and proliferation is aerobic glycolysis. Doumet Georges Helou et al. found that the lack of PD-1 led to significant up regulation of glycolytic genes (such as Hk1, Pkm and G6pdx), and increased glucose consumption, that is, blocking PD-1 can enhance glycolytic metabolism (32) (**Figure 2**).

PD-1/PD-L1 axis is mainly involved in TH1 cell reaction, and they are significantly expressed on activated antigen presenting cells (APCs) (33–36). After induction by the major histocompatibility complex (MHC), PD-L1 can bind to CD28 and CTLA-4 (inhibitory receptors) on the surface of activated T cells and then inhibit T cell activation and proliferation (37, 38). As one of the most important immunosuppression checkpoints, PD-L1 and PD-1 inhibit the overall activation signal through ligand-ligand-cis interaction. Studies have shown that in patients with active SLE, the normal process of PD-L1 expression is impaired. Blocking PD-1 can affect the disease activity in lupus mouse model, but the lack of PD-L1 did not cause SLE, but rather aggravated the systemic autoimmunity of lupus susceptible mice (39). In addition, in the knock-in mouse model that lacked ligand cis-action, the autoimmunity of the mice was greatly attenuated (11).

Previous studies have found that the expression of PD-L1 may decrease during the onset of systemic lupus erythematosus(SLE) (40). The interaction between PD-1 and PD-L1 reduces the autoimmunity of surrounding normal tissues, and the disruption of this interaction is involved in the pathogenesis of autoimmune diseases (41). The controlled PD-L1 expression also maintains immune tolerance by inhibiting the activation of T cells to prevent damage to the host immune system. Daisuke Sugiura et al. proposed an effective strategy to trigger PD-1 function by targeting the cis-PD-L1-CD80 complex, which potentiates the PD-1/PD-L1 interaction to inhibit autoreactive T cells, thereby alleviating autoimmune diseases (42).

## 4 Regulation of PD-1 and sPD-1 expression in SLE

In recent years, genetics and metabolomics studies have found that smoking, ultraviolet light, diet, microbial flora disorders, interleukin polymorphisms and predisposition are influencing factors for the pathogenesis and severity of SLE (43, 44). Inflammatory cytokines such as IL-6 and TNF-a can activate NF- κB pathway and increase the miR-34a expression on the surface of peripheral blood mononuclear cells (PBMCs), which then inhibits the expression of Foxp3 (It is expressed in a subset of Treg cells with specific transcriptional functions that maintain immune homeostasis) and disrupts the immune homeostasis (45). Taku Okazaki et al. found that PD-1-deficient mice produced self-reactive antibodies, which were generated by the dysregulated gut microbiota and induced germinal center (GC) T cells with proinflammatory activities (26). Bing Wan et al. reported that in the synovial fluid of patients, there was a soluble splicing variant of PD-1(PD- 1Δex3) that can antagonize the co inhibition function of PD-1 (46). Consistently, the latest research showed that SLE was characterized by abnormal type I IFN signal transduction. In the lupus mouse model, type III IFN λ (IFN- λ) can induce active TLR7 on B cells and promote the development of tissue inflammation (47–49). The factors that induce soluble PD-1 include IF-γ, IFN-λ, TNF α, and IL17A. The serum level of sPD-1 in SLE patients with high disease activity is significantly higher compared to those with low disease activity (17). sPD-1 could block the PD-1/PD-L1 interaction, significantly enhance T cell immunity, and lead to adverse disease outcomes. An *in vitro* cohort study also found that in SLE patients, the expression level of serum sPD-1 was significantly increased, and there was a significant positive correlation between the expression level and SLEDAI score. Thus, high expression level of sPD-1 can predict disease activity and prognosis (13).

The main feature of unstimulated lupus B cells and T cells is the increased expression of PD-1. After the stimulation with IL-2 for 48 hours, the ability of B-cells in SLE patients to up-regulate PD-L1 expression is significantly reduced (50).In the serum of SLE patients, although the number of B cells is significantly expanding and the transcription level is increasing, the effector molecules connecting B cells and PD-1 are phosphorylated by tyrosine kinase and PD-1 cannot be effectively expressed. Therefore, the major identified defect of CD8^+^T cells in SLE patients is the suppressed expression of PD-1 (51–53).

The polymorphism of the regulatory intron in PD-1 gene is associated with an increased risk of SLE (18, 54). It has been shown that PD-1 cross-linking can inhibit the proliferation of T cells and the production of cytokines in the serum of normal people and lupus patients (**Figure 1**). Jin Lin et al. explored the potential relationship between the recently discovered T-cell subpopulation PD-1^+^CXCR5^-^CD4^+^T peripheral helper cells (Tph) and the pathogenesis of SLE through cohort study.They found that the number of Tph cells was positively correlated with SLE disease activity (16).

## 5 PD-1 expression in SLE and its correlation with clinical outcome

**Figure 3:**
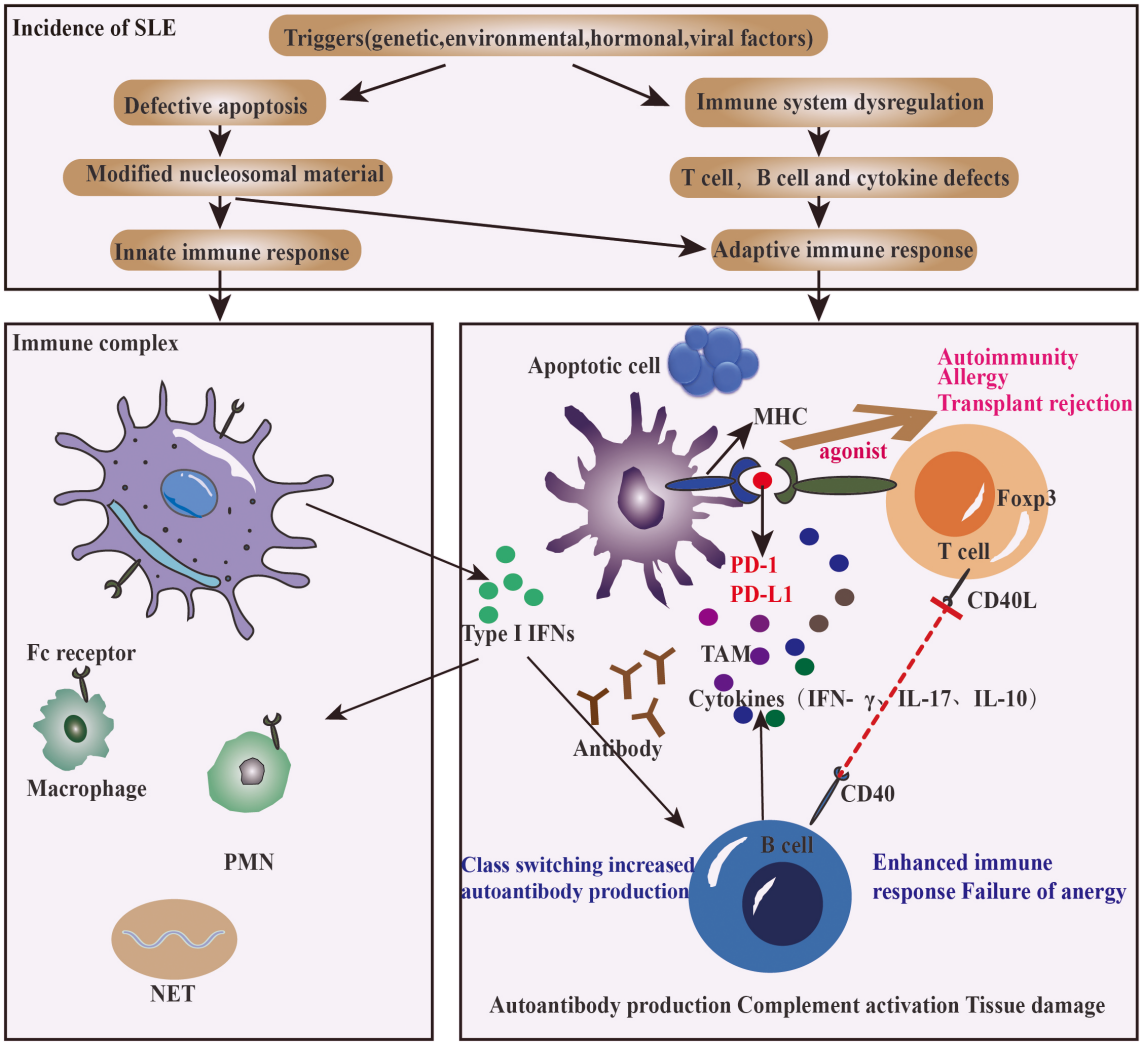
How PD-1 agonist improves the clinical outcomes of SLE.

Targeting PD-1/PD-L1 is a promising treatment for chronic autoimmune diseases (**Figure 3**). Some tumor cases showed that, after the use of PD-1 pathway inhibitors in cancer therapy, the original SLE disease activity or lupus nephritis attack was induced, including new onset of SLE (55, 56).

It is known that in SLE, tumor associated macrophages (TAM) disorder occurs when the activation of PD-1 by its ligand is affected or blocked (57, 58). As one of the heterogeneous cell populations, TAM can consume amino acids to secrete immunosuppressants, which can promote cell migration, survival and exocytosis (59). TAM is also closely related to the pathogenesis of SLE. If the immune checkpoint is selectively blocked, the killing of tumor cells by TAM will be enhanced, but it will also lead to autoimmune diseases such as SLE and rheumatoid arthritis (51).

In SLE, the chronic inflammatory mediators will drive the activation of FOXP_3_ HELIOS Tregs derived from the thymus, followed by the compensatory restoration of immune balance (23). Ricardo C Ferreira et al. showed that stimulating PD-1 with specific agonists could effectively inhibit autoreactive T cells and selectively restore the homeostasis of Tregs cell population (23), confirming that PD-1 plays a central role in SLE diseases.

In addition, PD-1 knockout mice showed lupus like autoimmune symptoms, accompanied by an increase in immunoglobulin and a decrease in complement; the exogenous addition of serum from active lupus patients led to the proliferation of T cells in these mice. This lupus murine model suggests that PD-1 is a negative effector of T cell activation, and the biological function of PD-1 antibody is to block PD-1 pathway and increase the activity of lupus (60).

The common cause of death in SLE patients is renal failure. According to statistical analysis, 5-20% of SLE patients developed end-stage kidney disease within 10 years after initial diagnosis (61). Therefore, early and accurate diagnosis and treatment of lupus nephritis is important for patient prognosis (61). A large amount of abnormally activated complement and immunoglobulin are deposited in foot cells, mesangial cells, renal tubular epithelial cells, renal macrophages, and stromal cells of the kidney (62). When PD-1 agonist was used to activate PD-1/PD-L1 axis, the affinity between PD-1 and reactive T cells was improved, and Th17 was significantly inhibited in multiple organs, which led to reduced inflammatory cytokines secretion in serum, such as IFN-γ, IL-17 and IL-10. The deposition of immunoglobulin in kidney and the level of urinary protein were also reduced and the survival time of mice was prolonged (63).

## 6 PD-1 is a promising biomarker to predict disease activity

The SLE Disease Activity Index (SLEDAI) has been the most widely used indicator to evaluate the disease activity of SLE. The complement level, anti-double-stranded DNA antibody titer and other parameters are included in the scoring standard (64). However, this indicator is still very limited in detecting the changes of SLE disease activity (65). Diogo Jesus et al. applied multivariate linear regression analysis to develop and verify a better SLE disease activity score system(SLE-DAS), which can be used for continuous measurement of the changes in SLE disease activity (66). In clinical practice, there is still a lack of a gold standard for the evaluation of SLE disease activity. The combination of laboratory indicators, clinical characteristics, and doctor’s overall impression of patient’ status (PGA score) is the basic judgment of SLE disease activity (67, 68). Essentially, all variables that are confirmed to be related to the disease are considered (67–69).

At present, PD-1 expression level is a widely used and accepted biomarker to guide immunotherapy (70). Active inflammatory factors in SLE patients can activate NF-κB and induce the overexpression of IFNs, which in turn up-regulates the downstream transcription factors such as STAT1, STAT4, STAT6 (71–73). STAT family is a crucial part of many signaling pathways that regulate cell growth and pathogen resistance (74). STAT pathway involves IL-6 (gp130) receptor family, which participates in regulating the differentiation of B cells and the expression of PD-1/PD-L1 (75). Jingwen Shi et al. reported that PD-1 blocks the up regulation of Tfh cells by inhibiting the PI3K activity downstream of follicle-guidance receptor CXCR5, which can control the position and function of Tfh cells in a co-stimulation dependent manner (76). The circulating Tfh-like T cells were amplified in the serum of SLE patients and altered the aberrant germinal center activity in these patients (77), suggesting abnormal homeostasis of T cells and B cells. The expression of PD-1 in Tfh cells is also related to SLEDAI and anti-double-stranded DNA antibody positivity, and hence the PD-1 expression can be used to measure disease activity (78).

It has been found that there is almost no expression of PD-1 or PD-L1 in the peripheral serum mononuclear cells (PBMC) of patients who meet the criteria of active SLE. However, during the remission of lupus, the expression of PD-1 and PD-L1 return to normal, indicating that there is a potential inverse correlation between the expression of PD-1, PD-L1 and disease activity. Therefore, we speculate that PD-1/PD-L1 expression can be used as a promising biomarker to predict disease activity (40).

## 7 PD-1 agonist monotherapy

Identifying common pathogenic pathways and corresponding biomarkers that link abnormal cell activity to disease activity are essential for finding new therapeutic targets. At present, the standard treatment of SLE is mainly non-specific immunosuppressant, which often causes side effects such as infection and leukopenia. Developing biological agents targeting specific targets or co-inhibition pathways is an effective way to reduce the side effects (79).

As an crucial immune checkpoint for T cell activation, PD-1/PD-L1 pathway can be effectively activated by PD-1 agonists to treat autoimmune diseases (80). The simulation of the interaction between PD-L1 and PD-1 on the surface of target cells and T cells can be used to precisely realize PD-1/TCR co-clustering on the T cell (81, 82). Adam P Curnock et al. have developed tissue specific PD-1 agonists that can avoid systemic immunosuppression and only inhibit local T cells, which can be used to treat highly chronic inflammatory diseases (80).

Zhao P et al. established three groups of models (spontaneous T1D model disease delay study, cyclophosphamide (CP) accelerated T1D model disease delay study and α PD-1 accelerates T1D mode disease delay research) and showed that PD-1^+^ not only helped to control autoimmune diseases, but also maintained the normal adaptive immunity of the body (31, 83, 84).Therefore, we speculated that PD-1 agonists might be able to achieve specific inhibition of highly activated immune cells through PD-1 inhibition signals.

Adam P Curnock et al. developed a PD-1 agonist targeting T cell receptor, called PD-1 ImmTAAI molecule, which can specifically promote the binding between PD-1 and PD-L1 on target cells while avoiding systemic immunosuppression. So far, the PD-1 agonists developed for autoimmune diseases are in Phase II and III clinical trials (**Table 1**). These drugs can stimulate the physiological immunosuppressive pathway and restore immune regulation, and are expected to become a new treatment scheme for autoimmune and autoimmune inflammatory diseases (80).

**Table 1:** PD-1 agonist antibody developed for autoimmune diseases registered in Insight Database.

Drug	R&D institutions	Phase	Global highest state time	Indication
Rosnilimab	AnaptysBio	II	2022-01-11	Alopecia areata
JNJ-67484703	Johnson & Johnson	I	2021-07-06	RA
Peresolimab	Eli Lilly and Company	II/I	2020-11-17	RA, Psoriasis
CC-90006	AnaptysBio Bristol-Myers Squibb Company Pharmaceutical company Celgene	I	2016-10-13	Plaque psoriasis
RTX-002	RubrYc Therapeutics	Pre-clinical	—	Autoimmune disease
PT627	Pandion Therapeutics Merck & Co., Inc.	Pre-clinical	—	Autoimmune disease

RA, Rheumatoid arthritis.

## 8 The combination of PD-1 agonist and low-dose Interleukin-2

For autoimmune diseases, the use of a single immune checkpoint inhibitor (ICI) alone is not as effective as a combination of several drugs (23). Thereby, the combination of drugs may provide a better treatment efficacy for SLE patients (85). By using flow cytometry and qPCR, He Hao et al. showed that the proportion of PD-1 ^hi^ T follicular helper (Tfh) cells (P<0.01) increased in the serum of SLE patients, while IL-2, the cytokine that induced T-follicular helper cells to transform into T-follicular regulatory (Tfr)cells, was low. Therefore, administration of low-dose IL-2 supplementation may be able to relieve SLE by rebalancing Tfh and Tfr cells (86).

As an important inflammatory factor, interleukin-2 (IL-2) has been extensively studied in immune regulation. The latest research shows that different doses of IL-2 have different effects on immune regulation. High dose of IL-2 can promote the differentiation and expansion of effector and memory T cells, while low dose of IL-2 mainly act on regulatory T cells(Treg cells) (87). In the *ex vivo* coculture assays of Tfh and B cells, IL-2 stimulation can cause the downstream transcription factors STAT3 and STAT5 to selectively bind to FOXP3 and BCL6 gene loci and inhibit H3K27me3, which further suppressed the plasma mother cells (86). In addition, Ricardo C Ferreira et al. have shown that the FOXP3^+^HELIOS^+^Tregs in the serum of SLE patients have demethylated FOXP3, resulting in increased number of CD4^+^FOXP3^+^cells. This CD4^+^FOXP3^+^state can be maintained by IL-2 induction (23).

Jing He et al. first proposed to use low-dose IL-2 to selectively regulate T cell subsets to achieve the goals of balancing immunity and treating SLE (88). The safety and effectiveness of using low-dose IL-2 to treat SLE and restore immune balance have been confirmed (89). *In vitro* experiments showed that when IL-2 was used to stimulate B cells in SLE, it was found that although the proliferation of B cells was weakened, the ability of IL-2 to up-regulate PD-L1 was also decreased. As a result, when low-dose IL-2 is used to treat SLE, adding PD-1 agonist at the same time may provide better therapeutic effect (50). Moreover, there was evidence showing that PD-1 signaling pathway can inhibit the production of IL-2 (90). When PD-1 agonist was used to stimulate experimental mice, the expression of IL-2 was decreased, and a negative correlation between the expression of PD-1 and the production of IL-2 was identified (91). Therefore, it is also essential to supplement low dose of IL-2 when using PD-1 agonist alone. Studies have shown that PD-1 expression increases on Treg in response to low-dose IL-2 treatment, which is crucial for maintaining the stable regulation of Treg. The data suggests that the combination of low-dose IL-2 immunotherapy and PD-1 agonist can selectively correct the defects of Treg *in vivo*, and inhibit the proliferation of pathogenic auto-reactive PD-1 Teffs. Pre-clinical research is currently ongoing and the outcomes are highly anticipated (23).

In summary, the key to the treatment of SLE is regulating Tregs homeostasis. IL-2 can induce the expression of PD-1 on Tregs, and PD-1 can regulate the proliferation of IL-2–induced Treg (92). The impairment of PD-1 inhibitory function in SLE has been confirmed, and low dose IL-2 can restore its function (93). Therefore, the combination of PD-1 agonist and low-dose IL-2 is a promising treatment option for SLE.

## 9 Biosafety issues and future prospects

Although there is a strong reason to develop PD-1 agonist as a therapeutic treatment for SLE, the research progress in this field is limited due to the following biosafety issues and off-target concerns (94). When the disease is in different active states, PD-1 agonists have different effects on the disease (95). In addition, the effect of PD-1 is closely linked with the patient’s gene expression profile, disease activity indexes, pharmacokinetics, and the binding affinity between drugs and receptors.

PD-1 agonist has been shown to effectively inhibit autoimmunity and is used to treat SLE, but it can also bring off-target effect and side effects. PD-1 agonist can inhibit the immune reaction to viral infection (96, 97). When cellular immunity, humoral immunity and innate immunity are suppressed, the body’s ability to fight off virus is weakened. Second, PD-1 agonists may increase the risk of cancer. Activation of the PD-1 pathway can inactivate tumor-infiltrating lymphocytes, thus evading immune surveillance (98–102). Finally, the activation of PD-1 pathway leads to immunosuppression, thereby reducing the effectiveness of vaccination (101).

Although a single PD-1-targeted agents may have significant efficacy for SLE, the results from PD-1 knockout mice suggest that combination therapy is still required. At the current research stage, low-dose IL-2 combined with PD-1 agonist has been confirmed to be effective in treating SLE in pre-clinical trials, and in-depth research is required for the selection of drugs in clinical treatment. Moreover, this combination is also an important supplement to the precise treatment of autoimmune diseases, helping to maximize the therapeutic effect and minimize the side effects.

## 10 Conclusions

As a chronic inflammatory autoimmune disease, SLE often affects multiple organs of the body. Its disease evaluation and disease relief have always been the research focus of clinicians and scientists. For a long time, the treatment of autoimmune diseases such as SLE mainly targets the auto-reactive lymphocytes and/or the auto-antibodies secreted by them, but the non-selective inhibition of adaptive immunity often yield low efficacy. Different from other negative regulators, PD-1 exists on activated T cells and B cells and has antigenic specificity. Therefore, it is a promising immune target for the treatment of SLE. We found that the expression and activity of PD-1 and its pathway are tightly related to the incidence and disease activity of SLE. In addition, further studies also showed that the soluble form of PD-1, sPD-1, was negatively correlated with the disease activity of SLE. Therefore, we propose that PD-1 and sPD-1 may be potential biomarkers for predicting SLE disease activity. Based on the above reasoning, we also propose that the application of PD-1 agonist might be an effective approach to treat SLE. However, single drug therapy may not achieve satisfactory response rate. According to the literature review and the latest research progress, the combination of PD-1 agonist and low-dose IL-2 may have a broader therapeutic prospect and advantages.

## Author contributions

BW wrote the first draft of the manuscript. CC contributed to conception, review and editing. All authors contributed to manuscript revision, read, and approved the submitted version.
